# 863. Concomitant Trauma Drives Benefit from Expanded Surgical Coverage in Burn Care

**DOI:** 10.1093/jbcr/irag033.333

**Published:** 2026-04-06

**Authors:** Stacey M Knowles, Jonathan E Schoen, M Victoria P Miles, Shana Lennard, Jeffrey E Carter

**Affiliations:** University Medical Center Burn Center, Louisiana State University Health Sciences Center, New Orleans, LA; UCI Health Regional Burn Center, New Orleans, LA; University Medical Center Burn Center, Louisiana State University Health Sciences Center, Department of Surgery, New Orleans, New Orleans, LA; LSU Health New Orleans/University Medical Center New Orleans, New Orleans, LA; University Medical Center Burn Center, Louisiana State University Health Sciences Center, New Orleans, LA

## Abstract

**Introduction:**

Fewer than 1.5% of U.S. hospitals house an ABA-verified burn center, and fewer than 0.2% of U.S. surgeons have burn-specific fellowship training. These workforce limitations are frequently cited as barriers to timely burn care. However, while delays to emergent surgery have been well studied in other surgical disciplines, less is known about how staffing models affect the timing of burn excision and operative access. This study evaluates the impact of transitioning from a single surgeon (SS) to a three-surgeon (3S) model on time to surgery and clinical outcomes within an ABA-verified burn and trauma center.

**Methods:**

In this retrospective study, burn admissions were compared across two time periods: SS coverage (03/30/2018–03/30/2020) versus 3S coverage (03/30/2023–03/30/2025). Patients >15 years old with thermal, chemical, contact, electrical, or mixed burns requiring excision were included. Data collected included concomitant trauma status, admission date, case request (CR) date, surgery time/date (TOR), and length of stay (LOS) normalized to percent total body surface area burned (%TBSA). Medians with interquartile ranges (IQR) were reported, and group differences were analyzed using the Mann–Whitney U test.

**Results:**

A total of 428 patients met inclusion criteria: 210 in the single-surgeon cohort (34 with trauma) and 218 in the three-surgeon cohort (37 with trauma). Among patients without concomitant trauma, no significant differences were observed in TOR (75 vs. 80 hours, *p*=.78), time to CR (*p*=.36), or LOS per %TBSA (*p*=.21). In contrast, among patients with concomitant trauma, the 3S cohort demonstrated significantly shorter TOR (127 hours [IQR 74–158] vs. 161 hours [IQR 89–225], *p*=.03) and time to CR (19 hours [IQR 10–145] vs. 58 hours [IQR 3–160], *p*=.03), with no differences in %TBSA (*p*=.80) or LOS per %TBSA (*p*=.20).

**Conclusions:**

Expanding from SS to 3S shorten the time to burn excision for patients with isolated burn injuries, suggesting that delays in this population are primarily influenced by diagnostic or logistical challenges rather than workforce capacity. In contrast, for patients with concomitant trauma, increased surgical staffing was associated with earlier CR and shorter TOR, reflecting improved efficiency in the management of complex, multi-system injuries. These findings highlight that workforce expansion may not uniformly accelerate burn excision but can meaningfully enhance care coordination and operative access in patients with combined burn and traumatic injuries.

**Applicability of Research to Practice:**

Further research is required to minimize diagnostic delays in burn care and explore site-specific barriers to reducing OR delays. Burn centers with a high incidence of trauma or MCIs could reduce treatment delays by increasing the number of burn surgeons on staff.

**Funding for the study:**

Spirit of Charity Foundation Burn Fund.

